# Initial acquisition and succession of the cystic fibrosis lung microbiome is associated with disease progression in infants and preschool children

**DOI:** 10.1371/journal.ppat.1006798

**Published:** 2018-01-18

**Authors:** Marianne S. Muhlebach, Bryan T. Zorn, Charles R. Esther, Joseph E. Hatch, Conor P. Murray, Lidija Turkovic, Sarath C. Ranganathan, Richard C. Boucher, Stephen M. Stick, Matthew C. Wolfgang

**Affiliations:** 1 Department of Pediatrics, University of North Carolina at Chapel Hill, Chapel Hill, North Carolina, United States of America; 2 Marsico Lung Institute, University of North Carolina at Chapel Hill, Chapel Hill, North Carolina, United States of America; 3 Department of Respiratory Medicine, Princess Margaret Hospital for Children, Subiaco, Australia; 4 Telethon Kids Institute, University of Western Australia, Perth, Australia; 5 Department of Respiratory Medicine, Royal Children’s Hospital, Parkville, Australia; 6 Murdoch Children’s Research Institute, Parkville, Australia; 7 Department of Paediatrics and Child Health, University of Western Australia, Perth, Australia; 8 Department of Microbiology and Immunology, University of North Carolina at Chapel Hill, Chapel Hill, North Carolina, United States of America; University of Washington, UNITED STATES

## Abstract

The cystic fibrosis (CF) lung microbiome has been studied in children and adults; however, little is known about its relationship to early disease progression. To better understand the relationship between the lung microbiome and early respiratory disease, we characterized the lower airways microbiome using bronchoalveolar lavage (BAL) samples obtained from clinically stable CF infants and preschoolers who underwent bronchoscopy and chest computed tomography (CT). Cross-sectional samples suggested a progression of the lower airways microbiome with age, beginning with relatively sterile airways in infancy. By age two, bacterial sequences typically associated with the oral cavity dominated lower airways samples in many CF subjects. The presence of an oral-like lower airways microbiome correlated with a significant increase in bacterial density and inflammation. These early changes occurred in many patients, despite the use of antibiotic prophylaxis in our cohort during the first two years of life. The majority of CF subjects older than four harbored a pathogen dominated airway microbiome, which was associated with a further increase in inflammation and the onset of structural lung disease, despite a negligible increase in bacterial density compared to younger patients with an oral-like airway microbiome. Our findings suggest that changes within the CF lower airways microbiome occur during the first years of life and that distinct microbial signatures are associated with the progression of early CF lung disease.

## Author summary

CF lung disease is characterized by persistent airway infection by complex microbial communities. These communities often consist of pathogens and endogenous microbes typically associated with the oral cavity. The development of these complex communities and their relationship to CF lung disease progression is unclear. To understand the evolution of the CF lower airways microbiome, we applied sensitive molecular detection methods to characterize the bacterial DNA sequences in bronchoalveolar lavage (BAL) samples obtained from clinically stable infants and preschoolers who underwent bronchoscopy. Our findings demonstrate that CF infants have relatively sterile lower airways with a progressive shift to a microbiome dominated by aerobic and anaerobic bacterial species commonly associated with the oral cavity. This initial acquisition of a lower airways microbiome was associated with a significant increase in bacterial burden and increased airway inflammation. Transition from an oral dominated to a pathogen dominated lower airways microbiome correlated with a further increase in inflammation and the onset of structural disease despite a negligible increase in bacterial density. Our findings suggest that oral microbes may play an important role in early CF airway disease and could potentially predispose subjects to subsequent infection by pathogens.

## Introduction

Cystic fibrosis (CF) is a multisystem genetic disease in which pulmonary manifestations account for the majority of morbidity and mortality. CF lung disease is characterized by thickened airway secretions, bacterial infection, and neutrophil dominated inflammation that leads to progressive airway destruction (bronchiectasis) and, ultimately, respiratory failure [1]. Traditional pathogens associated with CF lung disease include *Staphylococcus aureus* and *Haemophilus influenzae* in the first years of life, followed by an increasing prevalence of *Pseudomonas aeruginosa* in older children and adults [2].

Sensitive molecular based (16S rRNA gene sequencing) microbiome analysis of respiratory secretions from children and adults with CF suggest that infection is polymicrobial and often includes both traditional CF pathogens and aerobic and anaerobic bacteria typically found in the oral cavity [3–8]. Extended culture methods have confirmed that bacteria typically found in the oral cavity are present and viable in CF respiratory secretions, with densities similar to those of pathogens [9, 10] and higher than that seen in samples from healthy volunteers [11]. Oral bacteria have also been found in distal areas of the lung from a young CF patient at time of lobectomy [12]. However, contamination of lower airways samples by oropharyngeal secretions during collection remains a significant concern in airway microbiome research [13]. Consistent with this concern, it has been shown that oral bacteria are abundant in sputum and throat swabs from end-stage CF patients immediately prior to lung transplant, but airway samples obtained directly from the explanted lungs immediately after transplant are dominated almost exclusively by traditional CF pathogens [14].

Carefully controlled bronchoscopy studies indicate that the lower airways microbiome of healthy volunteers is similar in composition, albeit significantly lower in density, to that of the oropharynx [15–18]. The lower airways microbiome in healthy individuals is likely derived from normal microaspiration of upper airways secretions and not contamination during bronchoscopy [13, 19, 20]. In healthy individuals, oropharyngeal bacteria are presumed to be transient in the lower airways and their detection is unlikely to represent true colonization. As seen in microbiome studies of healthy porcine lungs, much of the bacterial DNA recovered by bronchoscopy is DNase sensitive, indicating that it is largely derived from bacteria that have been killed by lung defenses [21]. Increased DNA from oral bacteria in the lower airways, during disease, may reflect increased residence time due to defective clearance or diminished innate immune function rather than colonization. It has been proposed that exposure of the lower airways to anaerobes and/or oral bacteria, even if transient, is likely to influence lung physiology, local metabolites and mucosal immune homeostasis [20, 22, 23].

In several countries (United Kingdom, Germany, and most CF Centers in Australia) antibiotic prophylaxis is given to children with CF until they are two years of age [24], while other European countries treat any bacteria detected in cough-swabs during routine CF clinic visits [25]. In the United States prophylactic antibiotic therapy for infants and young children with CF is not recommended [26]; however, a recent clinical trial in the US and Canada showed that children less than 6 years of age spent an average of two months per year on antibiotic therapy for increased respiratory symptoms [27].

Airway inflammation in CF can be detected within the first few months to years of life, despite the absence of clinically diagnosed infection [28, 29]. Furthermore, increased inflammation and structural lung disease occurs in children with CF despite the use of prophylactic antibiotics [30–33]. The underlying cause of early airway inflammation in CF remains controversial and may reflect undiagnosed infection [28, 29, 34], altered immune function [35, 36], or a response to mucus obstruction [37].

To better understand the microenvironment in very young, clinically stable, CF subjects, we applied sensitive molecular detection methods to characterize the microbiome of the lower airways using cross-sectional BAL samples obtained from infants and preschoolers who underwent bronchoscopy as part of the Australian Respiratory Early Surveillance Team for Cystic Fibrosis (AREST CF) study. The early CF lower airways microbiome was analyzed in relation to antibiotic prophylaxis, BAL markers of inflammation and structural lung disease, measured by chest computed tomography (CT), to elucidate the evolution of the lower airways microbiome with respect to disease progression.

## Results

### Study population and control samples

To examine the early lower airways microbiome in CF, we obtained BAL samples collected during annual AREST CF study bronchoscopies from 46 CF subjects, ranging in age from approximately 3.5 months to five years with a median age of 1.95 years and an interquartile range (IQR) of 1.13–4.06 years. All subjects were diagnosed with CF through newborn screening. Patients were intubated during CT and bronchoscopy to control breathing and avoid aspiration. Per Australian clinical care standards, all children with CF are prescribed amoxicillin-clavulanic acid as antibiotic prophylaxis during the first two years of life. Thirty samples were obtained from subjects aged two years or younger. Seventeen samples showed clinically defined infection (i.e., BAL cultures showed a density of ≥10^5^ colony forming units/ml of a recognized pathogen). Twenty-three subjects were homozygous and 22 subjects were heterozygous for the F508del CFTR mutation. One subject had two other CFTR mutations. All subjects were seen routinely by a CF nutritionist and recommended a high fat diet with enzyme supplementation. Breastfeeding status was not collected.

For 25 patients, we processed two separate aliquots of pooled BAL collected from the right middle lobe (RML) for methods development. A single RML-derived BAL sample was studied from an additional 21 patients. Seven of the 46 subjects in this study had a second longitudinal BAL sample collected from the RML four to 22 months after the initial sampling. Longitudinal samples were included in observational analyses, while only a single sample per patient was considered in statistical analyses (see Methods). Clinical and study related data for all patients/samples is provided as Supporting Information (S1 Appendix). To control for background signal introduced during patient sampling, a saline wash was collected from two study associated bronchoscopes prior to BAL; bronchoscope washes were not available for all samples. Additionally, an aliquot of sterile water was included as a process control at the DNA extraction step. All patient and control samples were analyzed by bacterial 16S rRNA gene sequencing and quantitative polymerase chain reaction (qPCR) to determine bacterial identity and density, respectively.

### Composition of the early CF lower airways microbiome

DNA was extracted from all samples as previously reported for similar studies [14, 15]. Given that infant BAL is likely to have low biomass, we randomly selected a subset of patient samples for protocol development, using the method of Lundberg et al. as a starting point [38]. Empirical testing indicated that 25 cycles of initial amplification of the V4 16S rRNA gene region followed by 20 cycles of barcoding was necessary to generate sufficient 16S rRNA gene amplicons for sequencing. All samples, including controls were individually barcoded, pooled and sequenced simultaneously. After sequence processing and quality control, a total of 7.5 million sequences were classified into 423 Operational Taxonomic Units (OTUs).

**Fig 1:**
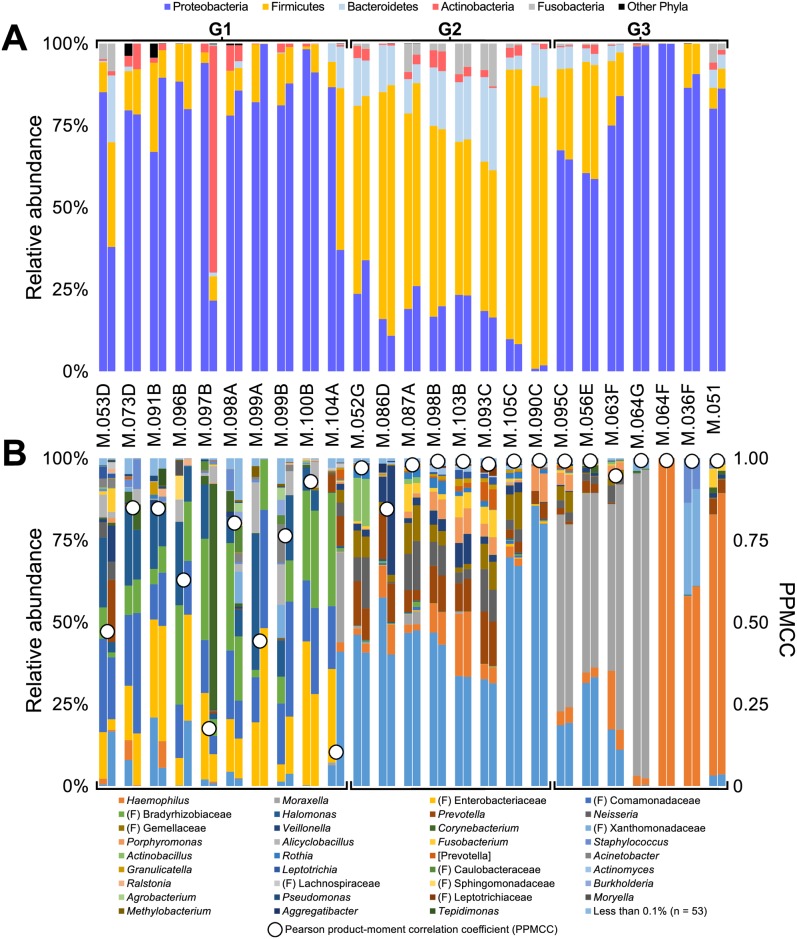
Taxonomic profile of replicate BAL samples. Relative abundance of bacterial taxa present in replicate BAL samples for each patient at the **(A)** phylum and **(B)** genus or lowest identifiable taxonomic level. Secondary axis **(B)** indicates the Pearson product-moment correlation coefficient (PPMCC) when comparing the proportion of sequences assigned to each taxon between respective replicates.

Given the potential for PCR bias in low biomass samples, we assessed the reproducibility of our methods by comparing the 16S rRNA gene sequence data for all 25 duplicate BAL aliquots (three were longitudinal), which matched the age distribution of the larger cohort (median, 1.86 years; IQR, 1.11–3.97). For analysis, these replicate samples were partitioned from the main sample set and rarefied to 6,000 sequences to retain all samples and represent their sequencing depth evenly. For the 25 duplicate BAL aliquots, the median Pearson product-moment correlation coefficient (PPMCC, *r*) between replicates was 0.98 (IQR, 0.81–1.00), at the OTU level. Community composition was highly similar between most replicates when OTUs were binned to both phylum (Fig 1A) and the lowest identifiable taxonomic level (Fig 1B), where the median PPMCC was 0.98 (IQR, 0.85–1.00) for the latter.

Non-metric multi-dimensional scaling (NMDS) analysis of the replicates showed that patient samples separated into discrete groups (S1 Fig). Those replicates with the lowest correlative values generally co-localized to the same group (Fig 1B, S1 Fig) and were subsequently determined to contain low biomass (S1 Appendix) and background sequences (see below). Comparison of the replicate samples suggested that our methodology was sufficiently robust, and that single BAL samples were likely to provide a reasonable description of the lower airways microbiome in our cohort.

### Distinct bacterial community structures define the early CF airways microbiome

**Fig 2:**
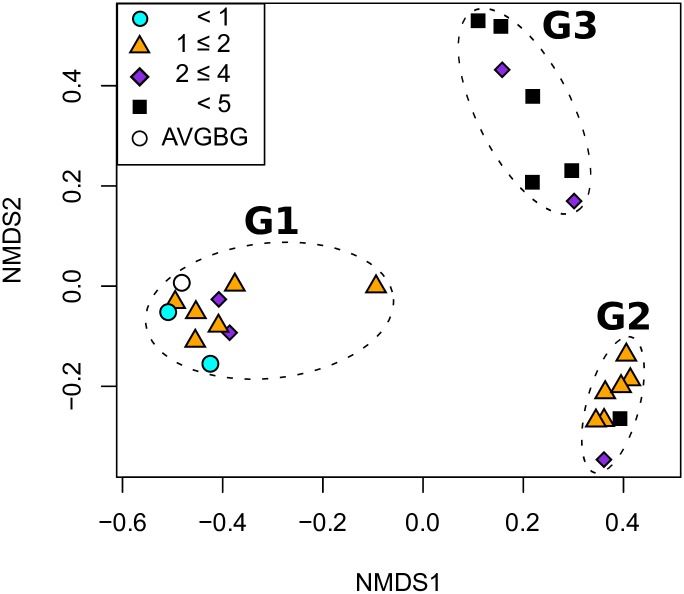
Merged sequence data for replicate BAL aliquots shows distinct sample groupings. Scaled Bray-Curtis distance NMDS performed on OTUs in merged replicate BAL samples (Stress: 0.043, non-metric fit *R*^*2*^ = 0.998). The average background community (AvgBG, white circle) was treated as an additional sample and included for comparison. Patient age at the time of sampling is denoted and groups are labeled and indicated by a dashed line.

Given the general agreement of the replicate samples, sequence data were pooled for each of the 25 duplicate BAL aliquots and rarefied to 11,000 sequences. Analysis of the combined aliquot data by NMDS confirmed that the samples separated into three discrete groups at the OTU level, designated as G1, G2 and G3 (Fig 2). G1 was composed of samples with the lowest replicate correlative values (median, 0.68; IQR, 0.44–0.87), while samples with the highest replicate correlative values segregated to G2 (median, 0.99; IQR, 0.98–1.00) and G3 (median, 1.00; IQR, 1.00–1.00). The groups also showed a trend with regard to patient age (Fig 2); G1 including the youngest patients (median, 1.08 years; IQR, 1.00–1.17), while G2 (median, 1.67 years; IQR, 1.20–1.96) and G3 (median, 4.09 years; IQR, 4.02–4.31) included increasingly older patients. Shannon diversity indices for each group were similar between G1 and G2; however, G3 was significantly lower than G2 (S2A Fig). The samples within G1 tended to have the largest differences in diversity scores between replicate pairs (median, 0.52; IQR, 0.27–0.71) as opposed to G2 (median, 0.20; IQR, 0.15–0.25) and G3 (median, 0.09; IQR, 0.03–0.29).

Analyses of the two bronchoscope washes and a process control (three background controls) showed high correlations with each other at the OTU and genus level (average *r* = 0.94, *r* = 0.94, respectively). As shown previously, the background signal originating from sample collection reagents and DNA extraction kits [39, 40] can dominate extremely low biomass samples and provide a signature for samples that otherwise lack appreciable amounts of bacteria [41]. Approximately 90% of the sequences in the three background controls were sourced from OTUs assigned to families Enterobacteriaceae, Bradyrhizobiaceae, and Comamonadaceae (S3A Fig). Previous sequencing studies have reported at least two of these taxa as contaminants derived from DNA extraction reagents [39, 40].

To determine whether background sequences contributed to the observed BAL OTU groups, we compared the average sequence signature for the background controls to the average for each of the observed sample groups. At the OTU level, the average background signal correlated highly with G1 (*r* = 0.81) and not with G2 or G3 (*r* = 0.02, *r* = -0.01, respectively). Further, the average background signal grouped with G1 samples, by NMDS (Fig 2). OTUs within G1 were dominated by Bradyrhizobiaceae, Comamonadaceae and Enterobacteriaceae, similar to the background controls (S3B Fig). In contrast, G2 was dominated by *Streptococcus* and G3 was dominated by *Haemophilus* and *Moraxella* (S3B Fig). Based on this analysis, we conclude that two potential bacterial community types are present in our early CF cohort, represented by G2 and G3, while G1 samples were largely explained by background signal and represent samples that lack an appreciable host-derived microbiome.

To determine whether these groups were maintained in a larger sample set, we expanded our analysis to include an additional 28 BAL samples (four of which were longitudinal) for which only a single aliquot was available. In addition, the average background signal was treated as an independent sample and included for reference. NMDS analysis of the complete cohort, at the OTU level, showed that many of the additional samples distributed closely with the previously defined groups (G1-G3). A subset of samples appeared to contain unique OTU distributions or a mixture of OTUs from multiple groups (S4 Fig). Additionally, the age distribution of the samples continued to exhibit a group associated trend (S4 Fig).

### Identification of early CF airways bacterial community determinants

**Fig 3:**
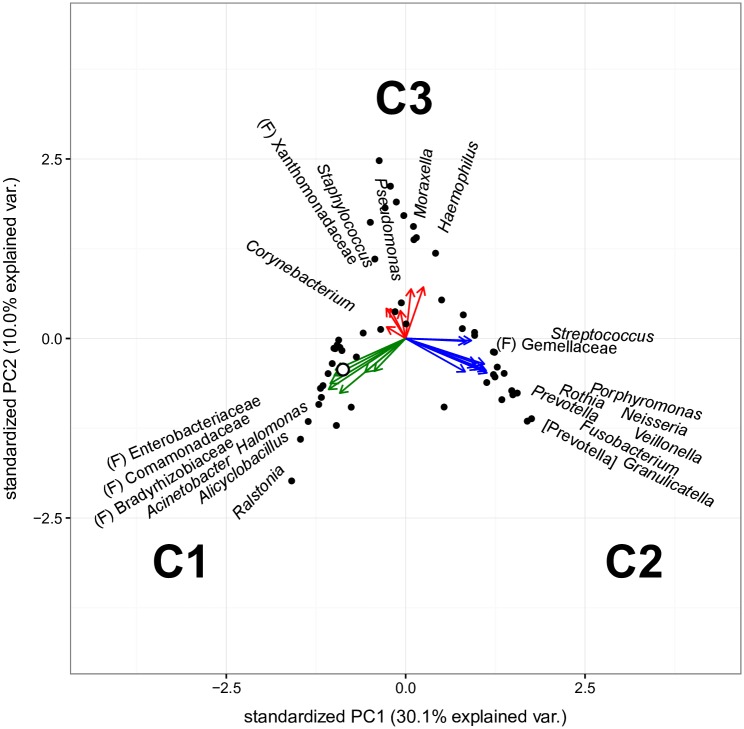
Principle component analysis (PCA) of the 23 most abundant taxa delineates three distinct bacterial associations or cluster types. Scaled PCA of the 23 most abundant taxa (representing ≥0.5% of the average relative abundance), for all RML samples and the average background community (white circle). Vector length and direction indicate the relative contribution of each taxon. Three associative groups of taxa are apparent. These clusters types can be summarized as background signal (C1, green), oral-like (C2, blue), or pathogen (C3, red) communities.

To identify the taxonomic associations that distinguish samples within our cohort, we conducted a principal component analysis (PCA). Previous studies have demonstrated that low biomass airway microbial community structure can be adequately defined by considering only the most abundant community members [41–43]. Therefore, to reduce complexity in our dataset, we considered taxa that contributed ≥0.5% of the average relative abundance across all samples. Based on this cut-off, 23 taxa accounted for >95% of all sequence data. Principal component analysis of all samples indicated that these 23 taxa separated into three distinct associations or clusters, designated C1, C2 and C3. (Fig 3). Cluster type C1 was defined by seven taxa; most often associated with the environment or abundant in our background controls (e.g., Enterobacteriaceae, Comamonadaceae, and Bradyrhizobiaceae). Further, the average background signal grouped with the C1 dominated BAL samples (Fig 3, white circle), indicating that these samples lack an appreciable lung derived microbiome. C2 was primarily defined by ten taxa typically associated with the oral cavity (e.g., *Streptococcus*, *Prevotella* and *Veillonella*). C3 was represented by six taxa, including those recognized clinically as pathogens in CF (e.g., *Haemophilus*, *Staphylococcus*, *Moraxella* and *Pseudomonas*). Shannon diversity for C1 and C2 samples were similar, while C3 values were significantly lower (S2B Fig).

To further examine and visualize the relationships between the members of these cluster types, we generated a correlation matrix for the 23 taxa used in the PCA, across all BAL samples (S5 Fig). As expected, there was a positive correlation between taxa within C1 (average *r* = 0.39±0.12) and C2 (average *r* = 0.43±0.06). However, taxa in C1 and C2 negatively correlated with each other (average *r* = -0.29±0.10), suggesting that the C1 and C2 clusters of bacteria tended to be mutually exclusive within our BAL samples. In contrast, taxa within C3 did not correlate with each other (average *r* = -0.02±0.03) or with taxa from C1 or C2 (average *r* = -0.08±0.11 and -0.10±0.05, respectively), indicating that members of cluster type C3 generally occur independently of each other consistent with the lower diversity ascribed to this cluster.

### Cluster types are associated with differences in age and bacterial density

**Fig 4:**
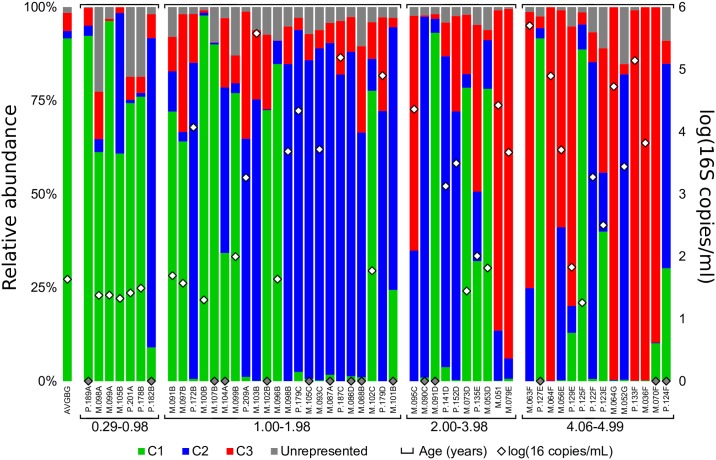
Relative abundance of each cluster type in BAL samples shows an age-associated trend from C1 to C3. Relative abundance of each cluster type suggests acquisition of an oral-like airway microbiome between one and two years of age. A gradual transition to a pathogen dominated community type begins around age four. Bacterial density is presented as the log of 16S rRNA gene copies/ml BAL for each sample on the secondary axis; samples with insufficient template for qPCR analysis are displayed as gray filled.

**Fig 5:**
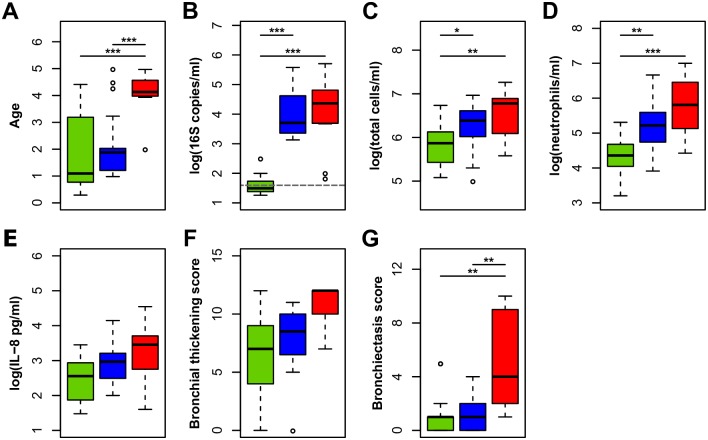
Cluster type is associated with bacterial density and clinical measurements of disease. **(A)** Age (years), **(B)** bacterial density (16S rRNA gene copies/ml BAL; average background signal denoted by dashed line), **(C)** total immune cell counts (TCC)/ml BAL, **(D)** total neutrophils/ml BAL, **(E)** IL-8 (picograms)/ml BAL, **(F)** bronchial wall thickening, and **(G)** bronchiectasis scores show significant changes based on cluster type. Significance between groups (C1: green, C2: blue, C3: red) were determined through Tukey’s HSD where single, double, and triple asterisks denote significance below 0.05, 0.005, and 0.0005, respectively. Outliers are defined as values above or below 1.5 times the difference in interquartile range above and below the quartiles within each group. Additional statistical analyses on these data can be found in S1 Table.

Given that >95% of sequences could be assigned to three associative clusters, we examined the BAL samples based on the relative proportion of each cluster (Fig 4). When viewed in this way, it was clear that some samples contained a mixture of cluster types, which helped describe the overall lower airways microbiome structure of the study cohort when observed through NMDS (S6 Fig). C1 dominated samples were most prevalent in the youngest patients (<1 year), whereas the C3 signature was generally associated with older subjects (>4 years) (Fig 4). The C2 BAL cluster type was most prevalent in the intermediate age group. While cross-sectional, these results suggest that the dominant cluster type may change in a temporal or age-associated manner. To test this hypothesis, each patient sample was defined as C1, C2 or C3 according to its dominant cluster type (S1 Appendix). Comparison of patient age based on these cluster designations showed that C1 dominated patients were generally younger, but not significantly different from C2. In contrast, C3 designated samples came from patients that were significantly older than both C1 and C2 patients (Fig 5A).

Samples assigned to C1 were associated with background signal, suggesting that they harboured extremely low levels of airways derived bacterial DNA. To determine if bacterial burden differed between samples based on the assigned dominant cluster type, we used qPCR to assess absolute bacterial 16S rRNA gene copies in each sample (Fig 4). Groupwise comparisons showed that C2 and C3 dominated samples were not different from each other, but had significantly greater bacterial densities than C1 (Fig 5B), which was similar to background (Fig 4). This further supports the conclusion that C1 dominated samples lack an appreciable lung-derived bacterial microbiome. As expected, there was a positive correlation between age and 16S rRNA gene copy number (*r* = 0.32; p≤0.05). Cluster assignments were further supported by clinical culture results, which showed that 10 of 12 subjects in C3 were infected with a known CF pathogen compared to 6 of 21 in C2 and 1 of 20 in C1 (S1 Appendix).

### Bacterial cluster type is associated with patient phenotype

We next evaluated the relevance of the dominant BAL cluster types to disease progression using BAL markers of inflammation and CT-defined structural lung disease as measures of disease severity (Fig 5C–5G, S1 Table). Patients with BAL samples defined as C1 had the lowest inflammation (total cell counts, total neutrophils and IL-8) and minimal to no detectible structural lung disease (bronchial wall thickening and bronchiectasis) based on CT scan. Patients with samples identified as C2 showed intermediate inflammation, with significantly higher total cell counts and total neutrophils compared to those designated as C1, but showed minimal structural change to the airways. While C2 samples were dominated by oral associated bacterial sequences, they often contained a small proportion of pathogen derived (C3) sequences (Fig 4). However, the presence of pathogen sequences in C2 designated samples did not account for the increase in inflammation (S7 Fig) in this patient group. Those patients with samples belonging to C3 exhibited further increases in both, inflammation and structural lung disease (Fig 5, S1 Table). To account for additional factors, including study site and antibiotic prophylaxis, we conducted multivariate analyses of the clinical data. When adjusted for these factors, the data indicated significantly higher BAL inflammation in patients with samples identified as C2 and C3 compared to C1 (S1 Table), and increased structural lung disease (bronchiectasis) in C3 designated patients compared to C1. Taken together, these results suggest that increasing disease severity correlates with changes in the lung microbiome.

To evaluate the specific contribution of oral flora and conventional CF pathogens, as distinct groups, to disease severity and progression, we used multiple regression analysis (S2 Table). When corrected for the presence of conventional pathogens, the relative abundance of oral-associated taxa (as a group) was significantly associated with increased bacterial 16S rDNA copy number, total cell counts and total neutrophils. These results demonstrate that the presence of an oral-like lower airways community is directly associated with early inflammation. In contrast, increasing relative abundance of conventional pathogens (as a group) was significantly associated with increased bacterial 16S rDNA copy number, measures of inflammation and structural lung disease (bronchial wall thickening and bronchiectasis scores). Further, the increasing relative abundance of both oral taxa and pathogens were significantly associated with measures of early inflammation when regression analyses were controlled for patient age (S2 Table).

### Longitudinal evaluation of community type

While the vast majority of samples in this study were cross-sectional, we evaluated seven subjects who had longitudinal BAL samples to test for temporal related changes in bacterial cluster type. Samples from five of the seven subjects did not show a change in cluster type over time. However, samples from two subjects showed a changed from an initial background dominated cluster type (C1) to an oral dominated cluster type (C2) (S4, S6 and S8 Figs). Interestingly, the single pathogen dominated longitudinal pair showed a shift from *Haemophilus* to *Moraxella* during the sampling interval (S8 Fig). Despite this genus level shift, the pathogen cluster type (C3) was maintained. While the number of longitudinal samples available for this study was limited, our analysis suggests that the relative composition of taxa within a given cluster type may be dynamic over time, but cluster typing designations are relatively stable. When cluster type did change within this subset of patients, it resulted in progression from C1 to C2.

## Discussion

### Methodological considerations

This study describes early changes in the CF lower airways microbiome in a unique study population, namely infants and preschool children who underwent bronchoscopy and CT scan at times of clinical stability. This young and clinically stable population showed absence of an appreciable lower airways infection in a subset of samples derived from the youngest subjects. Given the inherent low biomass in BAL samples from our cohort, extensive PCR amplification was needed to enhance sensitivity, which increased the risk of detecting background contamination [40]. Here, the background signal allowed for the differentiation and comparison of samples that lacked appreciable host derived bacteria, from those in which bacteria were present in appreciable quantities. The microbial signal present in BAL from the C1 subjects closely resembled the background detected in bronchoscope washes and processing reagents. The predominance of background sequences and low detectible bacterial biomass by qPCR (approximately 100 bacterial 16S rRNA gene copies/ml BAL on average) enhances our confidence that C1 designated samples came from patients that were not bacterially infected. This was consistent with clinical microbiology results, which showed that only one of the twenty C1 samples was culture positive for a pathogen. Importantly, the relative abundance of background sequences became negligible in the C2 and C3 samples, where non-background taxa dominated and bacterial biomass was increased by 3–4 orders of magnitude. Comparison of duplicate aliquots of the same BAL samples showed that our methodology was reproducible and that PCR bias was negligible. As expected, the greatest variability between replicates was seen in C1 samples that lacked appreciable patient derived bacterial DNA.

### Dynamics of microbial community structures and lung disease

Interestingly, we found that bacterial sequences detected in the lower airways samples of these CF infants and young children separated into three distinct cluster types. Further evaluation indicated that samples from our C1 group lacked a true lower airways bacterial community and represented an uninfected state. C2 samples harbored an actual microbial community resembling that found in the upper airway or oral cavity. We hypothesize that these samples represent the initial acquisition of a lower airways microbiome. Finally, we found that older subjects often harbored a lower airway bacterial community dominated by one or a few traditional CF pathogens.

We hypothesize that the increased abundance of bacteria typically found in the oral cavity in BAL reflects repeated micro-aspiration. Despite aspiration typically being more prevalent in infants less than one year of age, and bacterial communities being established in the oral cavity in the first few months of life [44, 45], subjects with BAL samples dominated by oral microbiota were mostly >12 months old in this study. Thus, we hypothesize that additional events early in life are necessary to establish lower airway niches capable of supporting the accumulation and persistence of oral microbes (e.g., decreased mucociliary clearance, increased mucus accumulation, and an anaerobic microenvironment). Given that bacterial density in BAL was similar in samples dominated by oral bacteria and pathogens, it seems possible that orally derived microbes may colonize or infect the lower airways; however, our study was not designed to distinguish airway colonization or infection from reduced clearance of aspirated bacteria. The oral community in these BAL samples included taxa identified in studies of older CF subjects by both molecular-based [3, 4, 8] and culture-based detection with densities approximating those of pathogens [11]. The role that these aerobic and anaerobic oral bacteria play in CF pathogenesis remains a subject of discussion. Recent functional studies have demonstrated that members of the oral microbiota may play an important role in conditioning the mucin rich lower airways environment for subsequent colonization by pathogens [22]. While many CF pathogens are incapable of efficiently metabolizing intact mucins for growth, consortia of oral microbes can use mucin carbohydrates as a primary nutrient source [22, 46]. Metabolites derived from degradation of mucins by these oral-derived consortia, support growth of traditional pathogens [22]. In addition, short chain fatty acids released by fermentation also contribute to inflammation [47]. As such, the oral dominated community type, detected in our study, may drive nutritional changes and inflammation in the lower airways environment, providing a potential mechanistic explanation for the increase in BAL markers of inflammation and the microbial succession seen in our young CF cohort.

Those subjects with BAL samples harbouring recognized pathogens had the highest markers of inflammation and overt structural lung disease. When present, pathogen sequences were at a higher relative abundance than other taxa, despite the BAL not having significantly greater microbial density than oral bacteria dominated samples. This dominance of pathogens is consistent with previous reports of decreased microbial diversity in oropharyngeal (OP) swabs across a wide age range [3, 48, 49]. Sputum based studies typically showed a progressive decrease in diversity with increasing disease [4, 50, 51]. In older patients, these findings may reflect increased antibiotic exposure [52] and/or once acquired, pathogen competition [53]. Here, progression of the airway microbiome and disease markers was heterogeneous, but generally changed with age. Multivariate analyses adjusted for age, indicated that the association of the pathogen dominated community type with increased neutrophil counts and bronchiectasis remained significant.

### Challenges of assessing the earliest CF airways microbiome

Although contamination of BAL with oral secretions can occur during bronchoscopy, this was minimized by placement of an endotracheal tube prior to the procedure. Importantly, contamination during the procedure does not explain the age dependent appearance of distinct community types, or the presence of lower airway inflammation with oral microbiota. We acknowledge that we did not have access to healthy non-CF infant control BAL samples to assess the normal lower airways microbiome or measure baseline inflammatory markers; however, given the invasive nature of bronchoscopy, collection of such samples would not be ethical. Further, the focus of this study was to elucidate the development of a lower airway microbiome in children with CF.

All children in this study were prescribed amoxicillin-clavulanic acid during the first two years of life, which could have affected our results, or delayed the onset of lower airways acquisition of oral microbes. Our multi-variate analyses corrected for this; however, we cannot accurately determine or predict the effect of early antibiotics has on presence of the lower airway microbiome in general. Antibiotics are prescribed frequently and early to infants and children with CF during times of increased respiratory symptoms [27] and antibiotic prophylaxis is recommended in young children with CF in several countries [24].

However, in the face of antibiotic prophylaxis a transition from background to an oral-like lower airways microbiome occurred, which may imply that bacteria are able to persist or accumulate in lower airways niches (e.g., mucus) despite antibiotic exposure. Further, adjustment for antibiotic prophylaxis in the multivariate analyses showed progressive disease in those dominated by oral bacteria at an age while children were still on prophylaxis.

True longitudinal studies of BAL microbiome in infants and children are difficult to perform but several groups have taken approaches to assess the development of the airway colonization in CF children. Hoen et al. examined the development of the gastrointestinal and respiratory microbiome in infants with CF, where respiratory samples were obtained by OP swab [54]. They showed a core microbial community spanning oral and intestinal samples that included genera detected in our study. For the gut microbiota, a predictive change occurred prior to onset of *P*. *aeruginosa* detection. However, as shown by clinical microbiology, the predictive value of OP swabs to BAL derived lower airways samples is modest [55, 56]. In our study, such comparisons of oral and/or stool microbiome to BAL microbiome was not possible as the AREST CF protocol does not include upper airway samples or stool collection. A more recent study compared the microbiome of OP and nasopharyngeal (NP) swabs to BAL in children with different underlying airway diseases. The study found that microbial community composition differed significantly between the sample types [57]. Nonetheless, the underlying microbial communities detected were predictive of disease state (i.e., prolonged cough, non-CF bronchiectasis, and children without lower airway infection).

Longitudinal comparisons of nasal microbiota have revealed that differences emerged within the first year of life in CF compared to healthy infants [58]. Comparison of the NP microbiome in infants with CF to healthy infants showed increasing separation of the microbial composition (e.g., increased *Staphylococcus* OTUs in CF) between the groups during the first six months of life [59]. Our data using BAL sampling in all subjects supports the premise that CF infants are initially uninfected. With time, our subjects exhibited a progressive increase in the density of oral bacteria in the lower airways, which ultimately changed to a pathogen dominated environment.

This conclusion is consistent with data from a small subset of patients in our study that had longitudinally collected BAL. Five of seven patients maintained the same microbial community type between sampling intervals (3.6 to 24 months), while two subjects progressed from an uninfected or background cluster type to an oral bacteria dominated community over time.

### Summary and implications

This study is unique in that it utilized lower airways samples obtained by bronchoscopy through an endotracheal tube from clinically stable subjects with CF ranging from infancy to preschool age. The study revealed that most CF subjects less than one year old had negligible bacterial densities in their BAL. Subsequently, a change in composition was seen with predominance of oral bacterial sequences in the 1–2 year olds and increasing presence of pathogens in samples from 3–5 year old subjects. The acquisition of an oral bacteria dominated lower airway microbiome and the succession to a pathogen dominated microbiome were each associated with increased markers of disease. Based on our analyses, we conclude that stratification of patients based on BAL cluster type was highly indicative of disease status. Testing the hypotheses of microbial community succession, the relevance of oral aspiration, and mucus plugging as a contributor to disease progression will require a larger longitudinal sample set.

## Methods

### Subjects

AREST CF conducts annual bronchoscopy and chest CT scans on infants and children diagnosed with CF when clinically stable [60]. In this study we included BAL samples from children who had undergone bronchoscopy and CT between 2011 and 2013. Subjects were enrolled in the CF clinics at Princess Margaret Hospital for Children, Perth, Australia and the Royal Children’s Hospital, Melbourne, Australia. Antibiotic prophylaxis with amoxicillin-clavulanic acid (15 mg/kg/day) is prescribed during the first two years of life to all children with CF in Perth and Melbourne. Subjects / BAL samples in this study were stratified to include a range from the earliest BAL to 5 years with and without infection on BAL diagnosed by clinical microbiology.

### Ethics statement

All BAL samples used in this study were collected under AREST CF protocols at Princess Margaret Hospital for Children, Perth, Australia and the Royal Children’s Hospital, Melbourne, Australia. AREST CF protocols were approved by Ethics Committees at both institutions. All samples were anonymized and stored for research purposes. The parents of participating children were informed of risks and given time to agree or decline voluntary participation prior to the start of all research procedures. Written parental consent was obtained for all subjects and included approval to share samples with other studies. The use of anonymized AREST CF BAL samples in this study was approved by the University of North Carolina at Chapel Hill office of Human Research Ethics.

### Bronchoalveolar lavage and CT scan

Bronchoscopy and CT scan procedures were performed as previously described [60]. Briefly, intravenous general anaesthesia with intubation was used to control breathing for the CT scan and to avoid contamination of the bronchoscope. BAL was performed with three aliquots of 1 ml/kg normal saline instilled into and aspirated from the right middle lobe (RML) and one aliquot into the most diseased lobe per CT-scan. The first aliquot from the RML was collected in a separate suction trap and sent to clinical microbiology lab for culture. The pooled 2^nd^ and 3^rd^ aliquots were collected into a single suction trap and stored frozen at -80°C in separate 1ml aliquots. These aliquots were subsequently used for assessment of inflammatory markers and microbiome analysis.

Volumetric CT scans were used for inspiratory assessment and either a 3-slice protocol at end expiration or volumetric expiratory assessment. The previously used semi-quantitative CF-specific CT scoring system measures gas trapping, bronchial wall thickening and bronchiectasis [30, 61]. Markers of lung inflammation in BAL included total and differential cell counts, and IL-8 measured by ELISA as done previously [62, 63].

### Microbiome analyses

The V4 region of the bacterial 16S rRNA gene was targeted for sequencing in a two-step preparation, using modified universal primers adapted from Lundberg et al. [38]. Bead-cleaned, equimolar concentrations of amplicons (approximately 450 base pairs in size) were sequenced on an Illumina MiSeq using a V2 paired-end 500 cycle kit. Raw sequence data was deposited in the European Nucleotide Archive (Study ID PRJEB13657). Read and data processing was performed in QIIME 1.8.0 and R version 3.2.3. Specific details of quantification, sequencing, and analysis are provided (S1 File).

### Statistical methods

Clinical data were archived prospectively in the AREST CF database. Data are reported as median and interquartile (25–75%) range using inflammatory markers and CT scores as continuous outcomes. To account for multiple sampling, patients with longitudinal samples had either time point 1 or time point 2 randomly selected to be included in all statistical comparisons. This random selection was performed once and maintained throughout all analyses (see S1 Appendix). Group comparisons were performed using Tukey’s HSD with a significance threshold of 0.05. Multiple regression analysis was performed in R version 3.3.3. Multivariate analyses used linear mixed effects models adjusted for age, antibiotic prophylaxis, and study site with significance at 0.05. Analyses were performed using Stata 13.0 (StataCorp LP) and JMP Pro 12.0.1 Software (SAS).

## Supporting information

S1 Fig Comparison of replicates by NMDS shows general reproducibility. Scaled Bray-Curtis distance NMDS was performed on OTUs in replicate samples (Stress: 0.136, non-metric fit *R*^*2*^ = 0.981). Vectors connect replicate pairs and are labeled with patient ID and PPMCC for each pair is indicated in parenthesis.
(TIF)

S2 Fig Shannon diversity. Shannon diversity index for **(A)** groups found in replicate analyses and **(B)** cluster designations from analysis of the total cohort. Significance between groups and clusters types were determined through Tukey’s HSD where single and double asterisks denote significance below 0.05 and 0.005, respectively. Outliers are defined as values above or below 1.5 times the difference in interquartile range above and below the quartiles within each group. Additional statistical analyses on these data can be found in S1 Table.
(TIF)

S3 Fig Taxonomic profiles of groups found by NMDS show similarities and differences compared to the background controls. Relative abundances of taxa within **(A)** each individual background control (water denotes the DNA extraction control; W1 and W2, represent independent bronchoscope washes) and **(B)** the average background (AVGBG) and average for each sample group defined by NMDS (Fig 2). G1 was highly similar in composition to AVGBG (*r* = 0.81), G2 was primarily characterized by *Streptococcus* OTUs, and showed a poor correlation with AVGBG (*r* = 0.02). G3 was mainly composed of *Moraxella* and *Haemophilus* OTUs, and negatively correlated with the background community type (*r* = -0.004).
(TIF)

S4 Fig NMDS of all samples show blended distributions between those previously defined. Scaled Bray-Curtis distance NMDS performed on OTUs in all samples, including average background (white circle) (Stress: 0.153, non-metric fit *R*^*2*^ = 0.977). Black vectors connect merged replicate samples depicted in Fig 2. Red vectors with arrows show longitudinal samples with time in years between sampling. Patient age at the time of sampling is indicated.
(TIF)

S5 Fig Heatmap of PPMCC values among the top 23 taxa. Heatmap of the correlative PPMCC values where community members were pre-grouped by cluster type, showing association (green) and dissociation (red) between groups. Each variable (taxon) are colored by cluster type (C1: green, C2: blue, C3: red).
(TIF)

S6 Fig Spatial distribution of all cohort samples analyzed by NMDS is explained by cluster type proportions. Scaled Bray-Curtis distance NMDS performed on OTUs in all samples, including average background (white circle) as in S3 Fig (Stress: 0.153, non-metric fit *R*^*2*^ = 0.977). Pie charts serve as sample points and illustrate the relative proportion of each cluster type within the sample. Vectors with arrows indicate temporal direction and join longitudinal samples.
(TIF)

S7 Fig The presence of pathogens in C2 dominated patient samples do not account for changes in disease severity. Patients designated as C2 dominated had varying proportions of C3 within their cluster profile. When the proportion of C3 was correlated with clinical measures of disease such as bacterial burden, total cells, total neutrophils, and IL-8, no significant correlations were found.
(TIF)

S8 Fig Cluster profiles show general stability with gradual change over time. Relative abundance of taxa in longitudinal samples for each patient (time in years below patient ID) at the **(A)** genus or lowest identifiable taxonomic level and **(B)** cluster type over time. An average of 0.85 years passed between each longitudinal sampling, but showed relatively stable designations over time with two transitions from a C1 to a C2.
(TIF)

S1 Table Association of dominant cluster types with clinical variables and bacterial density. (DOCX)

S2 Table Multiple regression analysis of cluster type association with clinical responses. (DOCX)

S1 File Detailed methods for 16S quantification, sequencing, and microbiome analyses. (DOCX)

S1 Appendix Patient/sample metadata and accompanying taxonomic information. (XLSX)
