# Supplementary figures and images for: Initial acquisition and succession of the cystic fibrosis lung microbiome is associated with disease progression in infants and preschool children
Source: PLoS Pathog. 2018 Jan 18;14(1):e1006798. doi: 10.1371/journal.ppat.1006798 (PMC5773228; doi:10.1371/journal.ppat.1006798)

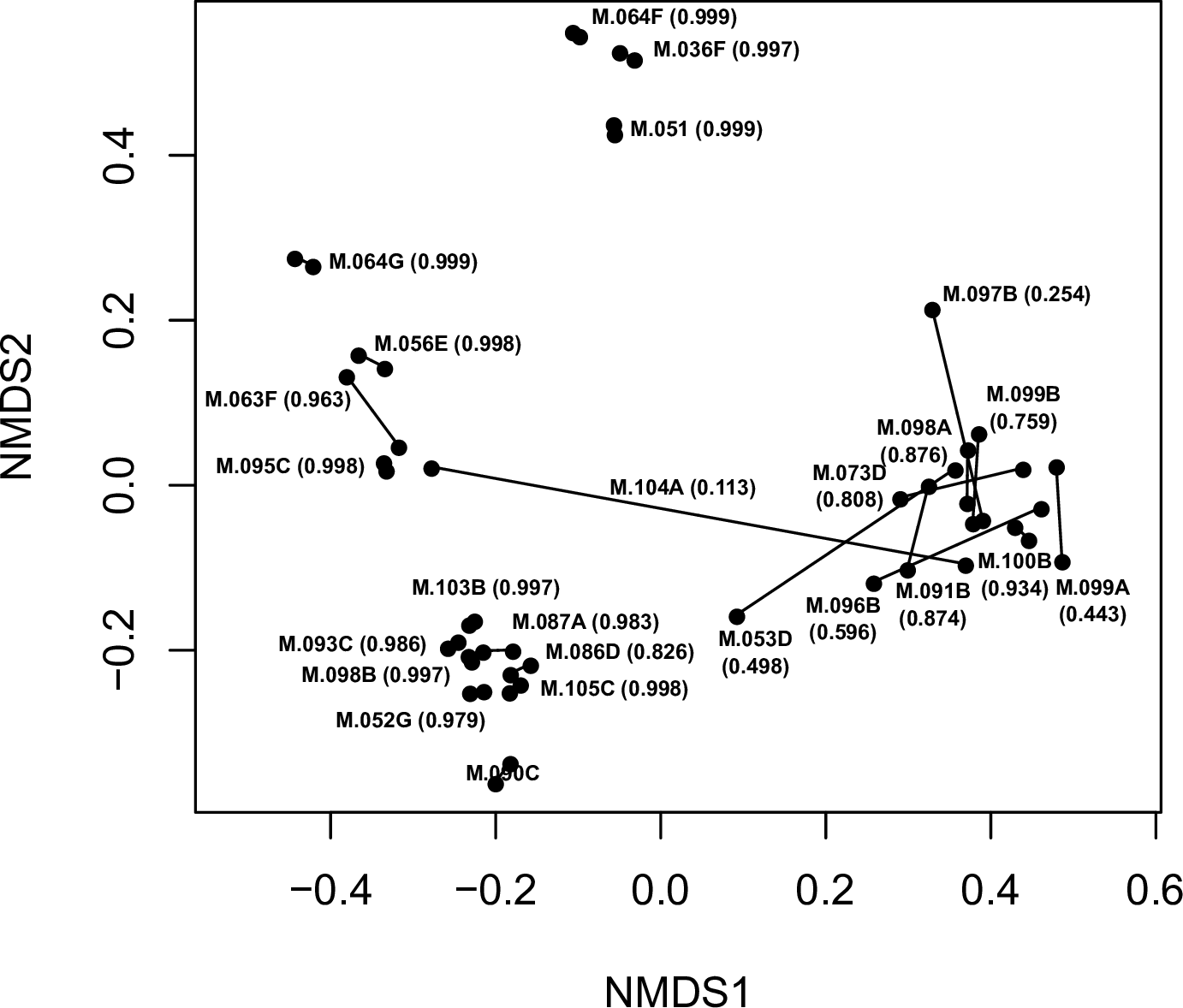

Supplement: S1 Fig — Scaled Bray-Curtis distance NMDS was performed on OTUs in replicate samples (Stress: 0.136, non-metric fit R2 = 0.981). Vectors connect replicate pairs and are labeled with patient ID and PPMCC for each pair is indicated in parenthesis. (TIF) [file ppat.1006798.s001.tif]

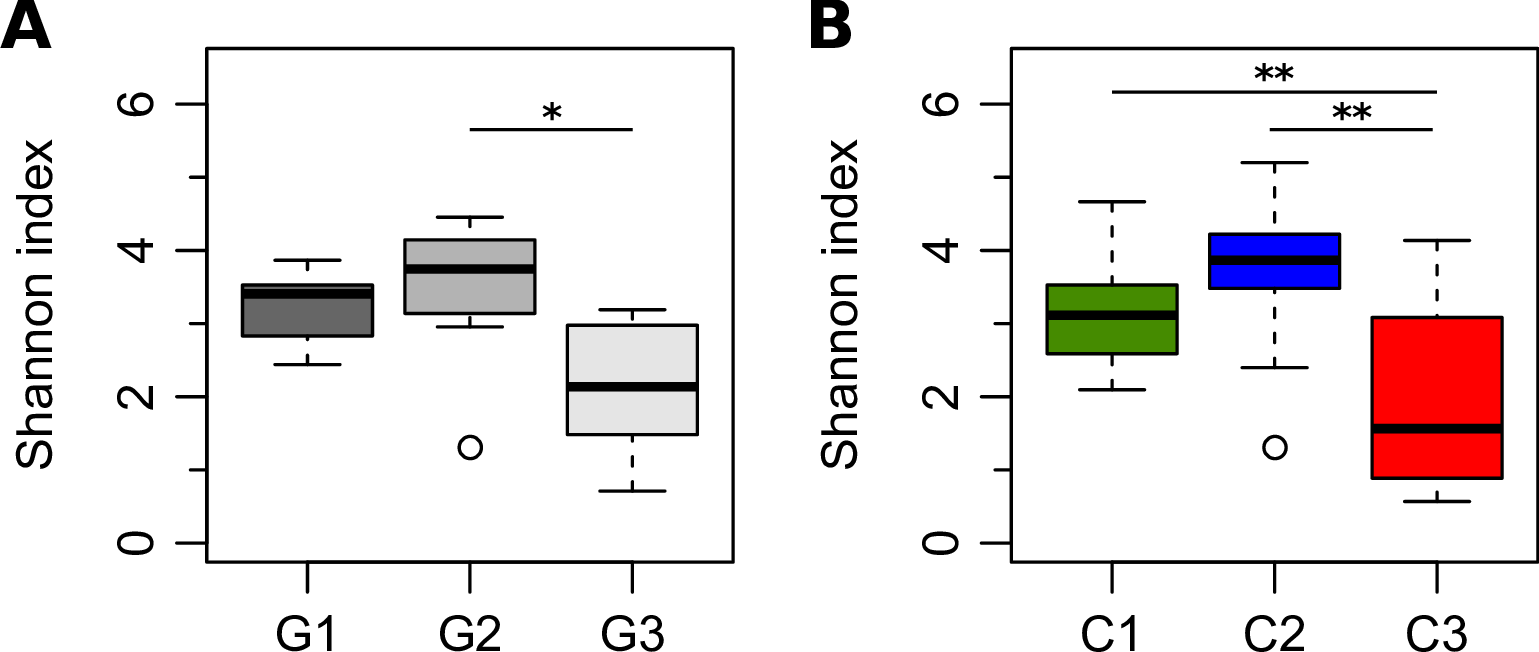

Supplement: S2 Fig — Shannon diversity index for (A) groups found in replicate analyses and (B) cluster designations from analysis of the total cohort. Significance between groups and clusters types were determined through Tukey’s HSD where single and double asterisks denote significance below 0.05 and 0.005, respectively. Outliers are defined as values above or below 1.5 times the difference in interquartile range above and below the quartiles within each group. Additional statistical analyses on these data can be found in S1 Table. (TIF) [file ppat.1006798.s002.tif]

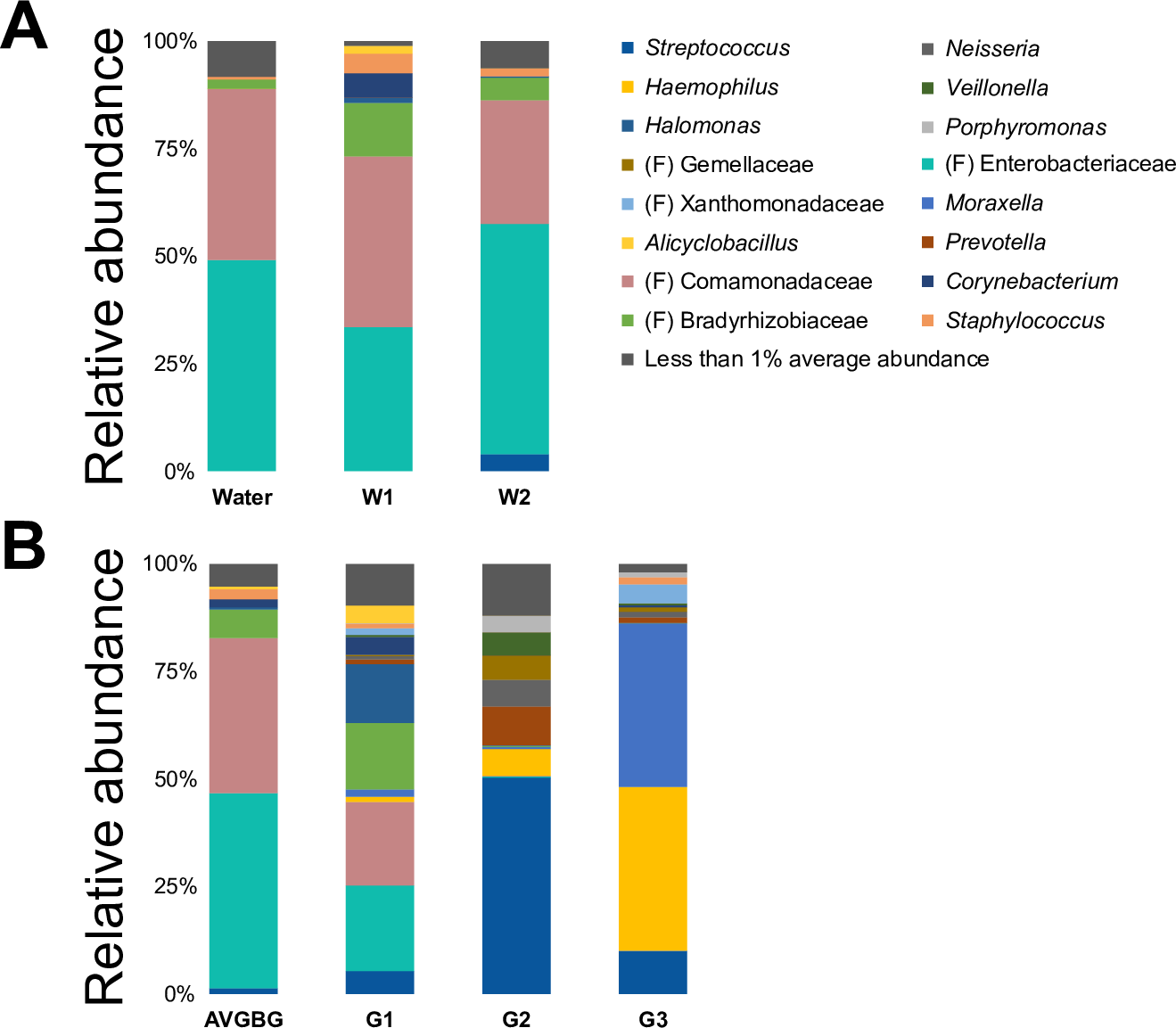

Supplement: S3 Fig — Relative abundances of taxa within (A) each individual background control (water denotes the DNA extraction control; W1 and W2, represent independent bronchoscope washes) and (B) the average background (AVGBG) and average for each sample group defined by NMDS (Fig 2). G1 was highly similar in composition to AVGBG (r = 0.81), G2 was primarily characterized by Streptococcus OTUs, and showed a poor correlation with AVGBG (r = 0.02). G3 was mainly composed of Moraxella and Haemophilus OTUs, and negatively correlated with the background community type (r = -0.004). (TIF) [file ppat.1006798.s003.tif]

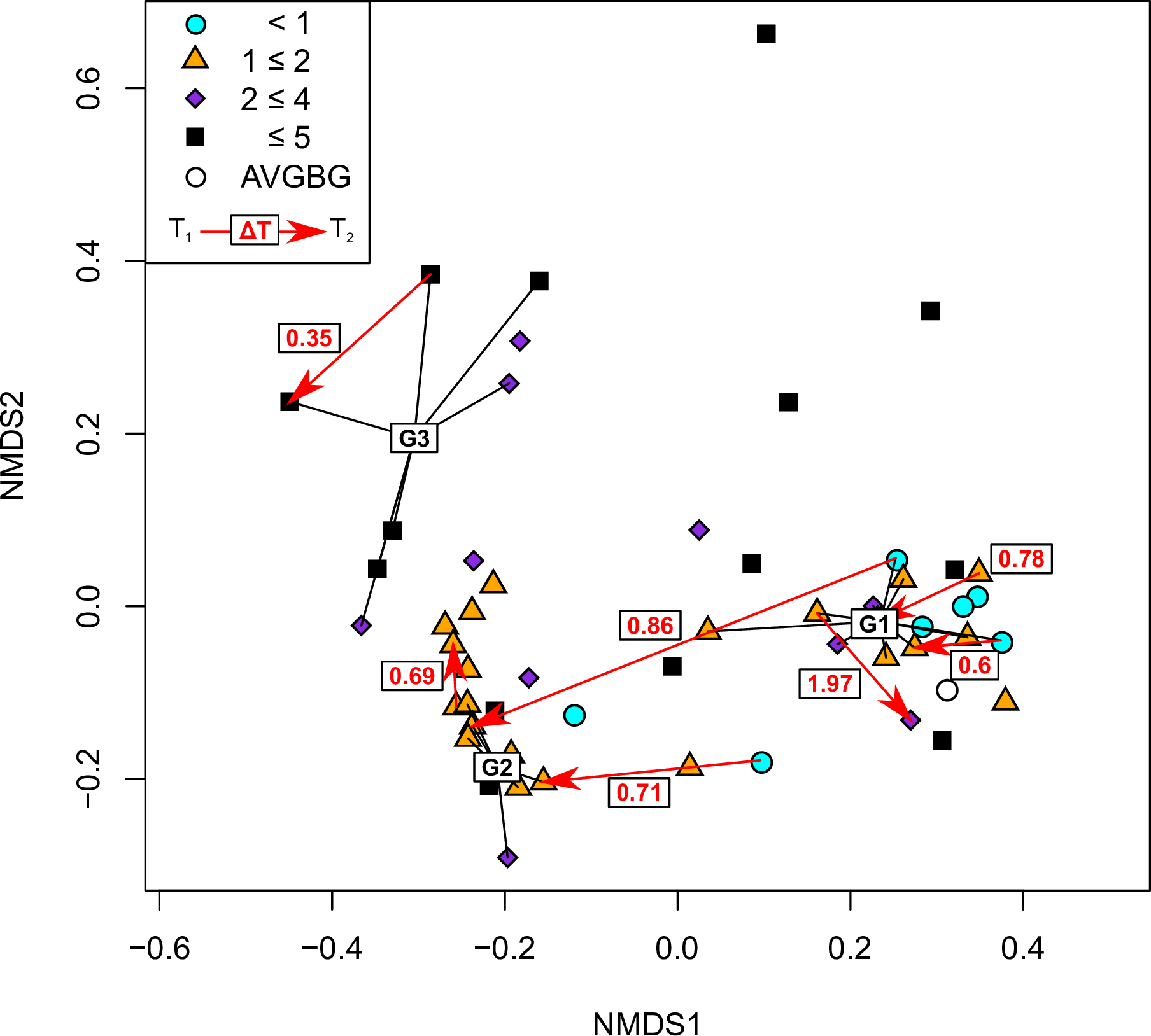

Supplement: S4 Fig — Scaled Bray-Curtis distance NMDS performed on OTUs in all samples, including average background (white circle) (Stress: 0.153, non-metric fit R2 = 0.977). Black vectors connect merged replicate samples depicted in Fig 2. Red vectors with arrows show longitudinal samples with time in years between sampling. Patient age at the time of sampling is indicated. (TIF) [file ppat.1006798.s004.tif]

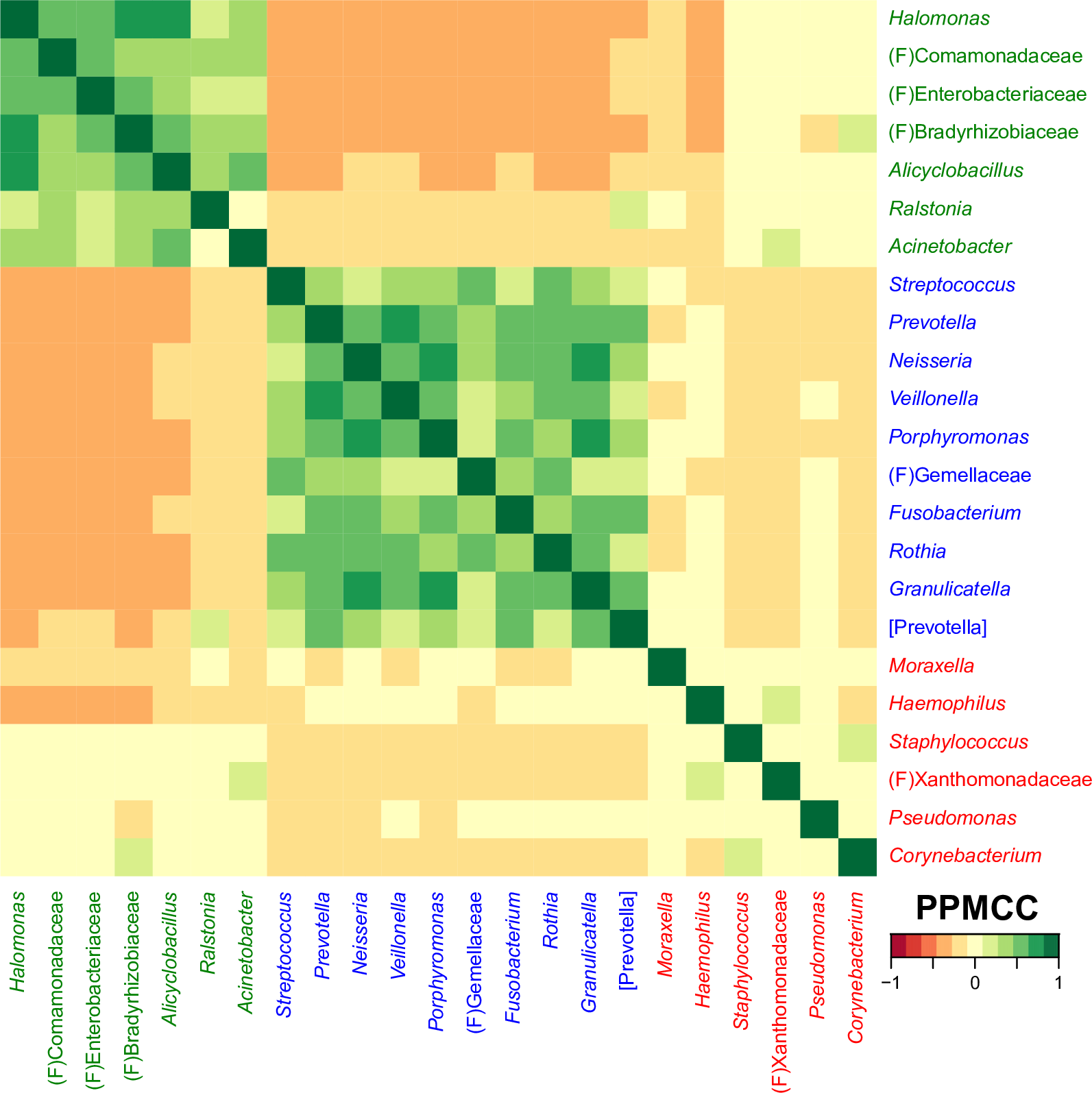

Supplement: S5 Fig — Heatmap of the correlative PPMCC values where community members were pre-grouped by cluster type, showing association (green) and dissociation (red) between groups. Each variable (taxon) are colored by cluster type (C1: green, C2: blue, C3: red). (TIF) [file ppat.1006798.s005.tif]

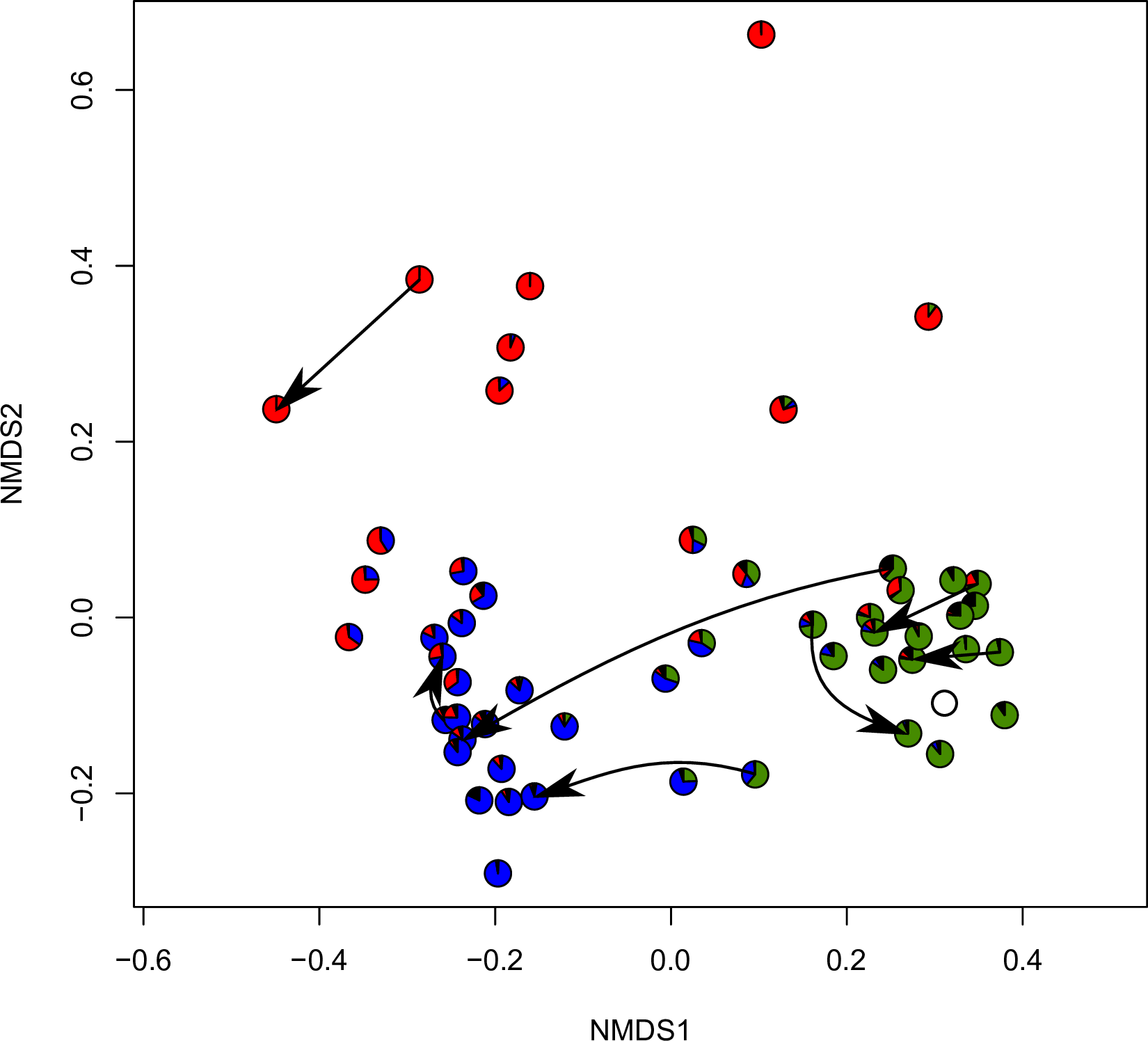

Supplement: S6 Fig — Scaled Bray-Curtis distance NMDS performed on OTUs in all samples, including average background (white circle) as in S3 Fig (Stress: 0.153, non-metric fit R2 = 0.977). Pie charts serve as sample points and illustrate the relative proportion of each cluster type within the sample. Vectors with arrows indicate temporal direction and join longitudinal samples. (TIF) [file ppat.1006798.s006.tif]

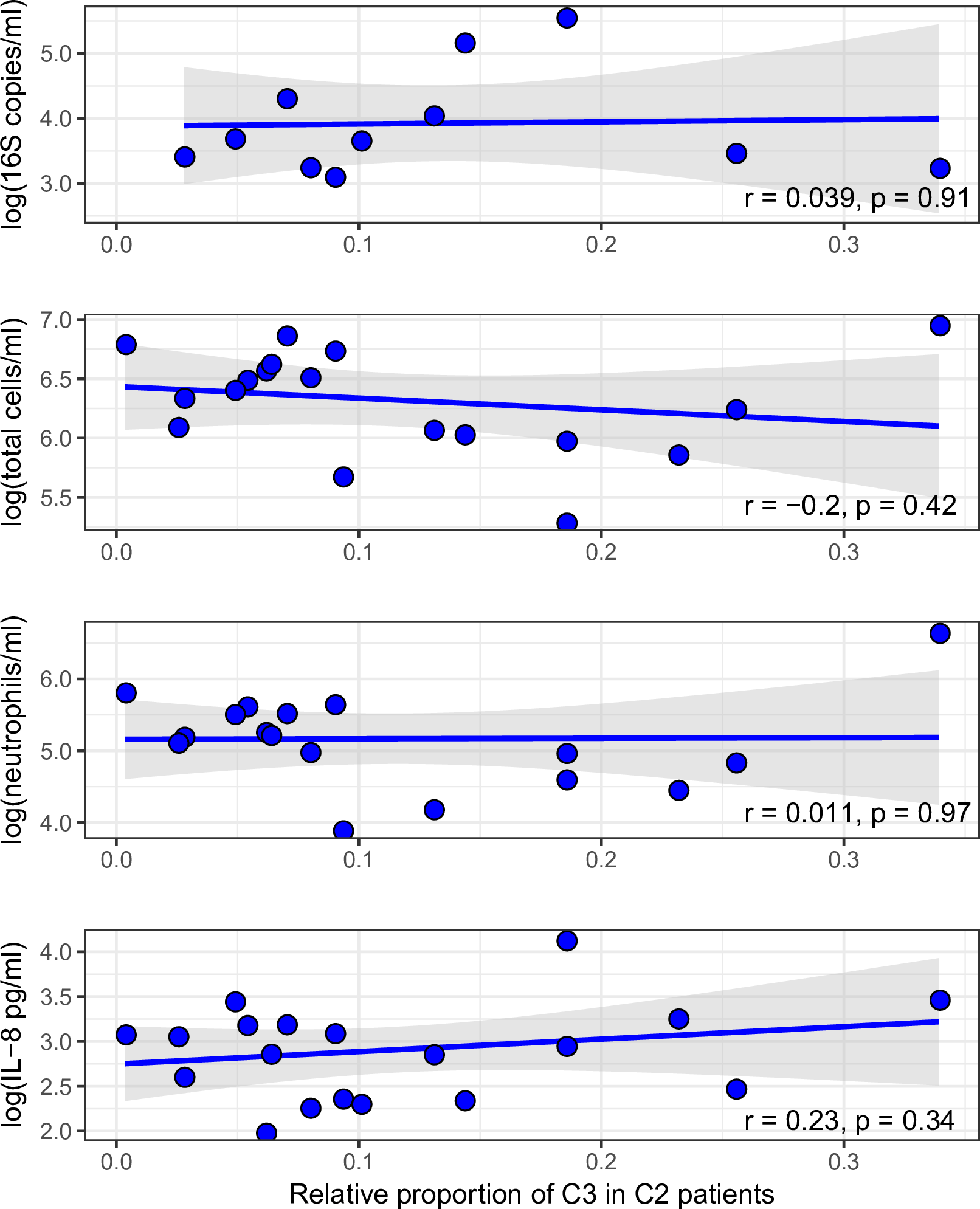

Supplement: S7 Fig — Patients designated as C2 dominated had varying proportions of C3 within their cluster profile. When the proportion of C3 was correlated with clinical measures of disease such as bacterial burden, total cells, total neutrophils, and IL-8, no significant correlations were found. (TIF) [file ppat.1006798.s007.tif]

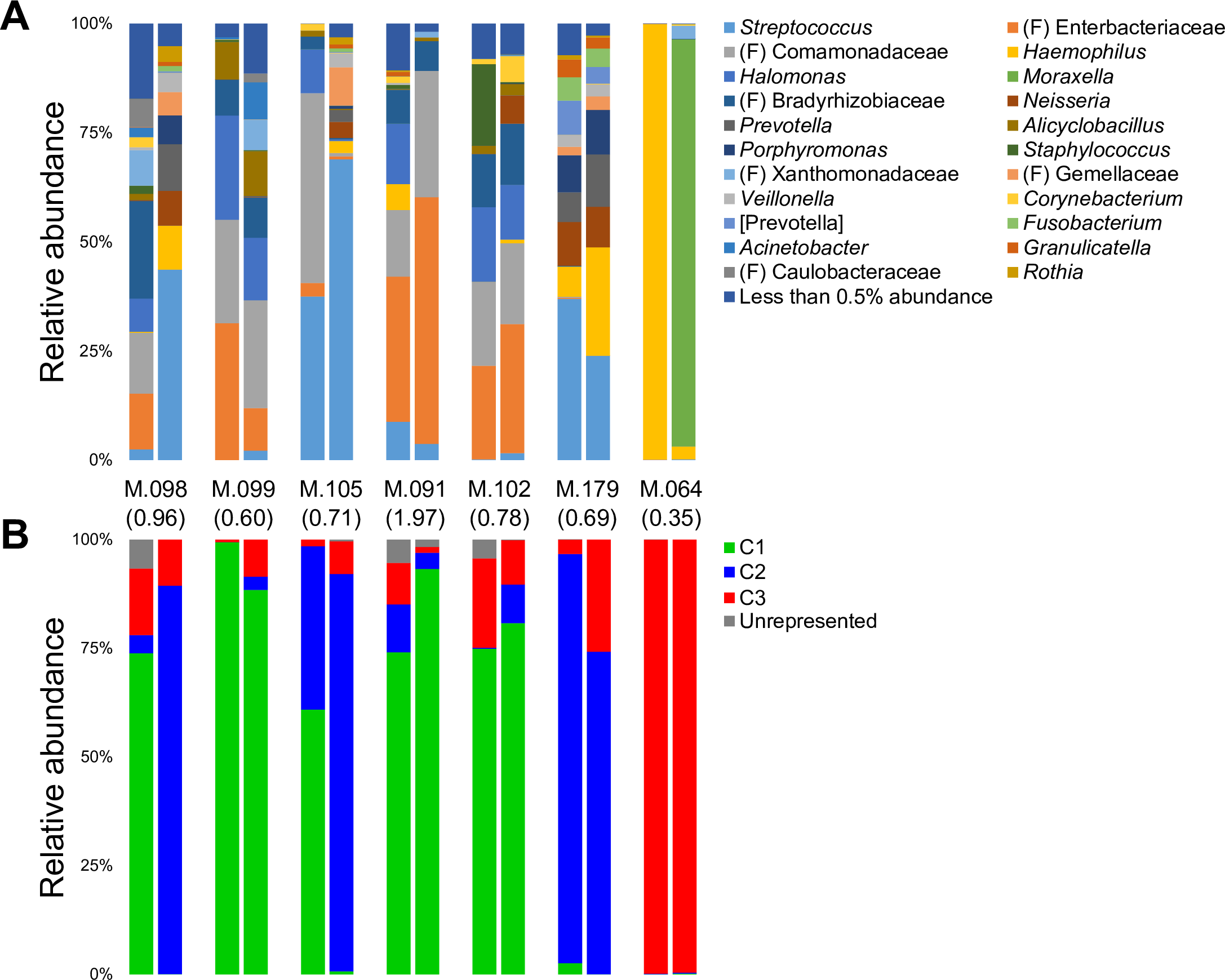

Supplement: S8 Fig — Relative abundance of taxa in longitudinal samples for each patient (time in years below patient ID) at the (A) genus or lowest identifiable taxonomic level and (B) cluster type over time. An average of 0.85 years passed between each longitudinal sampling, but showed relatively stable designations over time with two transitions from a C1 to a C2. (TIF) [file ppat.1006798.s008.tif]
