# Supplementary material for: Initial acquisition and succession of the cystic fibrosis lung microbiome is associated with disease progression in infants and preschool children
Source: PLoS Pathog. 2018 Jan 18;14(1):e1006798. doi: 10.1371/journal.ppat.1006798 (PMC5773228; doi:10.1371/journal.ppat.1006798)
Supplement: S1 Table — (DOCX) [file ppat.1006798.s009.docx]

| **S1 Table.** Association of cluster types with clinical variables and bacterial density. | | | | | | | | |
| --- | --- | --- | --- | --- | --- | --- | --- | --- |
|  | Cohort | C1 | C2 | C3 |  | MV model  co-efficient  C1:C2  C1:C3 | 95% conf. interval  C1:C2  C1:C3 | MV  p-value  C1:C2  C1:C3 |
|  | *n =* 46 | *n =* 16 | *n =* 19 | *n =* 11 |  |  |  |  |
| Age (years) ^b, c^ | 1.97  (1.11-4.07) | 1.06  (0.87-3.55) | 1.88  (1.18-2.58) | 4.17  (3.98-4.57) |  |  |  |  |
| qPCR (log copies) ^a, b^ | 3.27  (1.65-4.01) | 1.49  (1.38-1.69) | 3.67  (3.36-4.20) | 4.09  (3.68-4.65) |  |  |  |  |
| TCC (×10^4^) ^a, b^ | 140  (69-435) | 62  (24-101) | 243  (114-423) | 575  (123-747) |  | 0.47  0.46 | [0.19, 0.76]  [0.02, 0.89] | **0.001**  **0.039** |
| PMN (×10^4^) ^a, b^ | 9.4  (2.52-34.4) | 2.1  (0.96-4.5) | 16.4  (7.2-35.2) | 66.5  (11.7-285.5) |  | 0.80  1.07 | [0.31, 1.29]  [0.33, 1.80] | **< 0.001**  **0.005** |
| IL-8 (picograms) | 755  (215-2477) | 425  (90-1349) | 1190  (275-2185) | 2818  (565-3467) |  | 0.31  0.37 | [-0.11, 0.73]  [-0.29, 1.02] | 0.153  0.277 |
|  | *n =* 38 | *n = 13* | *n = 14* | *n = 5* |  |  |  |  |
| BWT | 8.5  (5-10.25) | 7  (3-9) | 8.5  (6.25-10) | 12  (10-12) |  | 2.02  3.18 | [-0.21, 4.26]  [-0.93, 7.29] | 0.076  0.129 |
| Bx ^b, c^ | 1  (0-2.25) | 1  (0-1) | 0.5  (0-1) | 4  (2-9) |  | -0.47  2.72 | [-1.83, 0.87]  [0.23, 5.20] | 0.487  **0.032** |
| Data presented as medians (with 25th-75th percentile below). Pairwise significant differences (bold) of the mean are indicated as superscript ‘a’, ‘b’, ‘c’ for comparisons C1:C2, C1:C3, and C2:C3, respectively. qPCR (16S copies), TCC (total immune cell count), PMN count (polymorphonuclear neutrophils; C2, n=17), and IL-8 are shown as per ml. TCC, PMN, and IL-8 values were not normally distributed; for analyses of significance these values were log transformed. Indicators of structural lung disease bronchial wall thickening (BWT) and bronchiectasis (Bx) are presented as scores. Multivariate model (MV) for change in outcomes using linear mixed effects models adjusting for age, antibiotic prophylaxis, gender and study site with C1 as comparator was used to determine the effect of microbial community types on clinical outcomes. | | | | | | | | |
