# Supplementary material for: Initial acquisition and succession of the cystic fibrosis lung microbiome is associated with disease progression in infants and preschool children
Source: PLoS Pathog. 2018 Jan 18;14(1):e1006798. doi: 10.1371/journal.ppat.1006798 (PMC5773228; doi:10.1371/journal.ppat.1006798)
Supplement: S2 Table — (DOCX) [file ppat.1006798.s010.docx]

| **S2 Table.** Multiple regression analysis of cluster association to clinical responses. | | | | | | |
| --- | --- | --- | --- | --- | --- | --- |
|  | qPCR | TCC | Neut. | IL-8 | BWT | Bx |
| C2 | **9.27 x 10^-9^** | **0.00681** | **0.00189** | 0.0643 | 0.327 | 0.471 |
| C3 | **5.43 x 10^-9^** | **0.00100** | **1.07 x 10^-6^** | **0.00667** | **0.0208** | **1.04 x 10^-4^** |
| St. Error | *0.734* | *0.453* | *0.661* | *0.623* | *3.31* | *1.89* |
| Adj. *R^2^* | *0.731* | *0.232* | *0.431* | *0.133* | *0.115* | *0.430* |
| C2 | **1.01 x 10^-8^** | **0.00583** | **0.00209** | 0.0646 | 0.405 | 0.407 |
| C3 | **8.84 x 10^-8^** | 0.130 | **4.97 x 10^-4^** | **0.0128** | 0.453 | **0.0101** |
| Age | 0.317 | **0.00767** | 0.0727 | 0.632 | 0.698 | 0.273 |
| St. Error | *0.733* | *0.435* | *0.661* | *0.623* | *3.31* | *1.89* |
| Adj. *R^2^* | *0.731* | *0.340* | *0.462* | *0.118* | *0.187* | *0.435* |
| Table is divided by two separate analyses; where C2 and C3 are used as dependent variables against clinical responses, and alternatively C2, C3, and Age. For each dependent variable, p-values are listed and when bold, are less than 0.05. Standard error and adjusted *R^2^* for each of the models are italicized. | | | | | | |
