# Supplementary material for: Initial acquisition and succession of the cystic fibrosis lung microbiome is associated with disease progression in infants and preschool children
Source: PLoS Pathog. 2018 Jan 18;14(1):e1006798. doi: 10.1371/journal.ppat.1006798 (PMC5773228; doi:10.1371/journal.ppat.1006798)
Supplement: S1 File — (DOCX) [file ppat.1006798.s011.docx]

**Detailed methods**

**Quantifying bacterial DNA**

Quantification of bacterial 16S rRNA gene copies in each sample was performed on a QuantStudio 6 Flex Real-Time PCR System (ThermoFisher Scientific) as previously described targeting the V1 and V2 16S rRNA gene with primers below purchased from Integrated DNA Technologies [1].

**qPCR amplification primers**

| Name | Sequence |
| --- | --- |
| V1_Forward  V2_Reverse  Probe | AGAGTTTGATCCTGGCTCAG  CTGCTGCCTYCCGTA  FAM-TA+ACA+CATG+CA+AGTC+GA-BHQ1 |

**Microbiome analyses**

Thawed BAL samples were centrifuged at 18000xg for 5 minutes and DNA from all samples was extracted using the Powersoil DNA Isolation Kit (MoBio Laboratories) as previously reported [1, 2]. Additional processing steps were previously described [3]. Briefly, the V4 region of the bacterial 16S rRNA gene was amplified from approximately 100ng of template DNA in a 50ul reaction with Phusion Hot Start II DNA Polymerase (ThermoFisher Scientific) using modified universal 515F and 806R primers (98°C for 30 seconds, 25 cycles of 98°C for 10 seconds, 52°C for 30 seconds, 72°C for 20 seconds, followed by 72°C for 5 minutes) [3]. Amplicons were cleaned with Axygen AxyPrep Mag PCR Clean-up Kit (Axygen Scientific) according to manufacturer’s instructions using 1:1 ratio. Bead-cleaned products were tagged with Illumina barcodes and Nextera adapters (98°C for 30 seconds, 20 cycles of 98°C for 10 seconds, 63°C for 30 seconds, 72°C 30 seconds, followed by 72°C for 5 minutes), purified again with Axygen beads as above, and visualized on a 1% agarose gel for appropriate fragment size (approximately 450 base pairs). A complete list of primers and associated sample IDs is available below. Products were quantified with Quant-iT Picogreen dsDNA Assay Kit (ThermoFisher Scientific), pooled in equimolar amounts (up to 20μl of product) and sequenced at the UNC High-Throughput Sequencing Facility on an Illumina MiSeq using a v2, paired-end, 500 cycle kit (Illumina Inc.). Raw sequence data was deposited in the European Nucleotide Archive (Study ID PRJEB13657).

Paired ends were joined using FastQ-Join (default parameters) within QIIME (version 1.8.0) [4]. Sequence data was demultiplexed with a custom AWK script designed to remove inline primer sequences and retain 16S rDNA sequences with greater than 85% of bases exceeding a Q score of 28. OTUs were determined via the open reference OTU picking workflow in QIIME, pick_open_reference_otus.py, where GreenGenes 13.8 served as a reference for both OTUs and taxonomy [5]. Chimeras were detected and removed from the data set using ChimeraSlayer within QIIME. After removing OTUs that were taxonomically unassigned (Kingdom: Unassigned), sequences were rarefied to different depths depending on the stage of downstream analysis. Individual replicates were rarefied to 6,000 sequences per sample, whereas merged replicates, negatives, and total cohort analysis were rarefied to 11,000 sequences per sample.

Bray-Curtis distances generated in QIIME were used as input for NMDS function metaMDS using the library “vegan” where centroid vectors were generated via function ordispider (https://CRAN.R-project.org/package=vegan) within R version 3.2.3 (<https://www.R-project.org/>). Superimposed pie charts as sample points on NMDS utilized the library “mapplots” with function add.pie (https://CRAN.R-project.org/package=mapplots). PCA was performed using base function prcomp with plotting function ggbiplot from library "ggplot2" [6]. Scatterplots of 95% confidence of correlation and significance were performed with ggscatter. Additional plots used R base package functions boxplot and heatmap, where cor was used to generate correlative values. Stacked bar charts were generated in Microsoft Excel where all autocorrecting features were turned off before importing data. Cosmetic modifications to figures were finalized in Inkscape (version 0.91, [http://www.inkscape.org](http://www.inkscape.org/)).

**Library amplification primers: primary template amplification**

| **806 Reverse 16S V4 frameshift primers**   \| Name \| Sequence \| Length \| \| --- \| --- \| --- \| \| 806R_f1 \| GTGACTGGAGTTCAGACGTGTGCTCTTCCGATCT NNNNN AC GGACTACHVGGGTWTCTAAT \| 61 \| \| 806R_f2 \| GTGACTGGAGTTCAGACGTGTGCTCTTCCGATCT NNTNNN AC GGACTACHVGGGTWTCTAAT \| 62 \| \| 806R_f3 \| GTGACTGGAGTTCAGACGTGTGCTCTTCCGATCT NNCTNNN AC GGACTACHVGGGTWTCTAAT \| 63 \| \| 806R_f4 \| GTGACTGGAGTTCAGACGTGTGCTCTTCCGATCT NNACTNNN AC GGACTACHVGGGTWTCTAAT \| 64 \| \| 806R_f5 \| GTGACTGGAGTTCAGACGTGTGCTCTTCCGATCT NNGACTNNN AC GGACTACHVGGGTWTCTAAT \| 65 \| \| 806R_f6 \| GTGACTGGAGTTCAGACGTGTGCTCTTCCGATCT NNTGACTNNN AC GGACTACHVGGGTWTCTAAT \| 66 \|   **515 Forward 16S V4 frameshift primers**   \| Name \| Sequence \| Length \| \| --- \| --- \| --- \| \| 515F_f1 \| GCCTCCCTCGCGCCATCAGAGATGTG TATAAGAGACAG NNNN NNNN GA GTGCCAGCMGCCGCGGTAA \| 67 \| \| 515F_f2 \| GCCTCCCTCGCGCCATCAGAGATGTG TATAAGAGACAG NNNN T NNNN GA GTGCCAGCMGCCGCGGTAA \| 68 \| \| 515F_f3 \| GCCTCCCTCGCGCCATCAGAGATGTG TATAAGAGACAG NNNN CT NNNN GA GTGCCAGCMGCCGCGGTAA \| 69 \| \| 515F_f4 \| GCCTCCCTCGCGCCATCAGAGATGTG TATAAGAGACAG NNNN ACT NNNN GA GTGCCAGCMGCCGCGGTAA \| 70 \| \| 515F_f5 \| GCCTCCCTCGCGCCATCAGAGATGTG TATAAGAGACAG NNNN GACT NNNN GA GTGCCAGCMGCCGCGGTAA \| 71 \| \| 515F_f6 \| GCCTCCCTCGCGCCATCAGAGATGTG TATAAGAGACAG NNNN TGACT NNNN GA GTGCCAGCMGCCGCGGTAA \| 72 \|   **Amplification primers: secondary template amplification and sequencing**  **Forward Illumina adapter – Forward MTFS annealing**  AATGATACGGCGACCACCGAGATCTACAC-GCCTCCCTCGCGCCATCAGAGATGTG  **Reverse MT-FS-annealing – Illumina barcode – Reverse Illumina Adapater**  TAGAGCATACGGCAGAAGACGAAC-XXXXXXXXX-CTCGTGTGCAGACTTGAGGTCAGTG  **Illumina Nextera P1 Sequencing primer**  GCCTCCCTCGCGCCATCAGAGATGTGTATAAGAGACAG  **Sample mapping information**  SampleID Illumina barcode   \| PIC.123e12.R01.24FEB12A1 \| TTACCGACG \| \| --- \| --- \| \| MIC.087A.BALRAW.9.2.12A10 \| ATTGGACAC \| \| PIC.129e12.R01.15JAN12A2 \| TCGCATGGA \| \| MIC.056E.BALRAW.27.3.12A3 \| AGCGAACCT \| \| MIC.056E.BALRAW.27.3.12A4 \| AGCTTCGAC \| \| MIC.064F.BALRAW.14.2.12A5 \| GTCAGCCGT \| \| MIC.064F.BALRAW.14.2.12A6 \| TCCAGATAG \| \| MIC.086D.BALRAW.24.4.12A7 \| GAGAGTCCA \| \| MIC.086D.BALRAW.24.4.12A8 \| GCTCACAAT \| \| MIC.087A.BALRAW.9.2.12A9 \| TTGACGACA \| \| MIC.088B.BALRAW.28FEB12B1 \| CTTAGAACG \| \| MIC.105B.BALRAW.1.3.12B10 \| CGGTTCACA \| \| MIC.093C.BALRAW.23FEB12B2 \| CGATAGGCC \| \| MIC.093C.BALRAW.28FEB12B3 \| GCTATATCC \| \| MIC.096B.BALRAW.23.2.12B4 \| GTCTTCAGC \| \| MIC.096B.BALRAW.23.2.12B5 \| TAGACACCG \| \| MIC.097B.BALRAW.2.5.12B6 \| TCAGCTGAC \| \| MIC.097B.BALRAW.2.5.12B7 \| TAAGTCGGC \| \| MIC.098A.BALRAW.10.11.11B8 \| GCTCCTTAG \| \| MIC.098A.C.BALRAW.10.11.11B9 \| ATGGCCTGA \| \| PIC.133F12.R01.21SEP12C1 \| TTGCAAGTA \| \| MIC.064G.BALRAW.19.6.12C10 \| CCTAGTAAG \| \| PIC.133F12.R04.21SEP12C2 \| CTAGGATCA \| \| PIC.179D12.R01.19OCT12C3 \| TATGAACGT \| \| PIC.179D12.R04.19OCT12C4 \| CTTGTGCGA \| \| MIC.052G.BALRAW.19.6.12C5 \| CACGATGGT \| \| MIC.052G.BALRAW.19.6.12C6 \| ACGTGCCTT \| \| MIC.063F.BALRAW.3.7.12C7 \| TGAACTAGC \| \| MIC.063F.BALRAW.3.7.12C8 \| TATTCAGCG \| \| MIC.064G.BALRAW.19.6.12C9 \| TAATCGGTG \| \| MIC.090C.BALRAW.19.6.12D1 \| GCGTCCATG \| \| MIC.104A.BALRAW.21.6.12D10 \| CGTAAGATG \| \| MIC.090C.BALRAW.19.6.12D2 \| CTGTTACAG \| \| MIC.098BD3.1 \| ACGATCATC \| \| MIC.098BD3.2 \| GTAACGGCT \| \| MIC.100B.BALRAW.4.10.12D5 \| CCATGCTTA \| \| MIC.100B.BALRAW.4.10.12D6 \| GTACGCACA \| \| MIC.103B.BALRAW.25.10.12D7 \| TTAGAGCCA \| \| MIC.103B.BALRAW.25.10.12D8 \| ATAAGGTCG \| \| MIC.104A.BALRAW.21.6.12D9 \| AGTGGCACT \| \| PIC.124F12.R01.30NOV12E1. \| CCAGAAGTG \| \| PIC.178B11.R01RAW.09SEP11E10 \| CTACTAGCG \| \| PIC.124F12.R04.30NOV12E2 \| TAGCGTTCC \| \| PIC.201A12.R01.14DEC12E3 \| GTGAGTCAT \| \| PIC.201A12.R04.14DEC12E4 \| TGGTCCTAC \| \| MIC.095C.BALRAW.20.11.12E5 \| TACGCGTAC \| \| MIC.095C.BALRAW.20.11.12E6 \| GAGCCATCT \| \| MIC.099B.BALRAW.6.9.12E7 \| CGTCCGTAT \| \| MIC.099B.BALRAW.6.9.12E8 \| GATACGTTC \| \| MIC.102B.RMLRAW.29.11.12E9 \| CAGCTGGTT \| \| MIC.107B.RML.7.2.13F1. \| TTAAGCGCC \| \| MIC.091D.LL.8.10.13F10 \| CCTGCGAAG \| \| MIC.107B.LL.7.2.13F2 \| TTGTAGCCG \| \| PIC.187C13.R01.12JUL13F3 \| TCTGTAGAG \| \| PIC.187C13.R04.12JUL13F4 \| CTATTAAGC \| \| MIC.079E.LL.21.5.13F5 \| CTCTGAGGT \| \| MIC.079E.RML.21.5.13F6 \| CAGGATTCG \| \| PIC.209A13.R01.15NOV13F7 \| TCACTGCTA \| \| PIC.209.A13.R04.15NOV13F8 \| ACATGTCAC \| \| MIC.091D.RML.8.10.13F9 \| ATTCTGCCG \| \| MIC.101B.LL.27.8.13G1 \| TACACGCTG \| \| PIC.152.D12.R01.24FEB12G10 \| TGCATACAC \| \| MIC.101B.RML.27.8.13G2 \| ACGCAATGT \| \| MIC.102C.LL.10.9.13G3 \| GCTCGAAGA \| \| MIC.102C.RML.10.9.13G4 \| AGACGTTGC \| \| PIC122F12.R01RAW.13JAN12G5 \| TAGAGCTGC \| \| PIC.125.F12.R01.29FEB12G6 \| GGTAACCTC \| \| PIC.127.E11.R01.11NOV11G7 \| GACTTCATG \| \| PIC.135.E11.R01.02DEC11G8 \| CTGCATACT \| \| PIC.141.D11.R01.21OCT11G9 \| TAAGGCATC \| \| PIC.172.B11.R01.26AUG11H1 \| AGTATTCGC \| \| MIC.053D.BALRAW.6.12.11H10 \| TTCGCAGAT \| \| PIC.179.C12.R01.10FEB12H2 \| GCACCTGTT \| \| PIC.182.B12.R01.13JAN12H3 \| CTCATGGTA \| \| PIC.189.A11.R01.04NOV11H4 \| ACTAGTTGG \| \| MIC.036F.BALRAW.15.12.11H5 \| GCGGACTAT \| \| MIC.036F.BALRAW.15.12.11H6 \| ATCGCTTAA \| \| MIC.051.1.9.11H7 \| TCAGGACGT \| \| MIC.051.1.9.11H8 \| GCATTACTG \| \| MIC.053D.BALRAW.6.12.11H9 \| GCTATGGAA \| \| MIC.091B.BALRAW.20.10.11I1 \| GATTGTGCA \| \| MIC.105B.BALRAW.15.11.2012I10 \| AGCCTCATG \| \| MIC.091B.BALRAW.20.10.11I2 \| AACTCCTGT \| \| MIC.099A.BALRAW.2.2.2012I3 \| TAGAAGGCT \| \| MIC.099A.BALRAW.2.2.2012I4 \| GACTAGTCA \| \| MIC.070F.LL.29.10.13I5 \| GGATACTCG \| \| MIC.070F.RML.29.10.13I6 \| CCGACATTG \| \| MIC.073D.BALRAW.26.7.12I7 \| TCGTGACGC \| \| MIC.073D.BALRAW.26.7.12I8 \| GGCCTATAA \| \| MIC.105B.BALRAW.15.11.2012I9 \| GTAGCACTC \| \| MIC.095B.BALRAW.13.12.2011J1 \| CTAAGACGT \| \| PIN.139A14.W01.31JAN14 \| CGTGCACAA \| \| PROCESS.CONTROL \| TGTAACGCC \| \| PIC.197C14.W01.04APR14 \| ATGCGAGAC \| \| WATER \| TAGTAGCAC \| | | | | | |  |  |
| --- | --- | --- | --- | --- | --- | --- | --- | --- | --- | --- | --- | --- | --- | --- | --- | --- | --- | --- | --- | --- | --- | --- | --- | --- | --- | --- | --- | --- | --- | --- | --- | --- | --- | --- | --- | --- | --- | --- | --- | --- | --- | --- | --- | --- | --- | --- | --- | --- | --- | --- | --- | --- | --- | --- | --- | --- | --- | --- | --- | --- | --- | --- | --- | --- | --- | --- | --- | --- | --- | --- | --- | --- | --- | --- | --- | --- | --- | --- | --- | --- | --- | --- | --- | --- | --- | --- | --- | --- | --- | --- | --- | --- | --- | --- | --- | --- | --- | --- | --- | --- | --- | --- | --- | --- | --- | --- | --- | --- | --- | --- | --- | --- | --- | --- | --- | --- | --- | --- | --- | --- | --- | --- | --- | --- | --- | --- | --- | --- | --- | --- | --- | --- | --- | --- | --- | --- | --- | --- | --- | --- | --- | --- | --- | --- | --- | --- | --- | --- | --- | --- | --- | --- | --- | --- | --- | --- | --- | --- | --- | --- | --- | --- | --- | --- | --- | --- | --- | --- | --- | --- | --- | --- | --- | --- | --- | --- | --- | --- | --- | --- | --- | --- | --- | --- | --- | --- | --- | --- | --- | --- | --- | --- | --- | --- | --- | --- | --- | --- | --- | --- | --- | --- | --- | --- | --- | --- | --- | --- | --- | --- | --- | --- | --- | --- | --- | --- | --- | --- | --- | --- | --- | --- | --- | --- | --- | --- | --- | --- | --- | --- | --- | --- | --- | --- | --- | --- | --- | --- | --- |
|  |  |  |  |  |  | |  |

**References**

1. Bassis CM, Erb-Downward JR, Dickson RP, Freeman CM, Schmidt TM, Young VB, et al. Analysis of the upper respiratory tract microbiotas as the source of the lung and gastric microbiotas in healthy individuals. MBio. 2015;6(2):e00037. doi: 10.1128/mBio.00037-15. PubMed PMID: 25736890; PubMed Central PMCID: PMCPMC4358017.

2. Goddard AF, Staudinger BJ, Dowd SE, Joshi-Datar A, Wolcott RD, Aitken ML, et al. Direct sampling of cystic fibrosis lungs indicates that DNA-based analyses of upper-airway specimens can misrepresent lung microbiota. Proc Natl Acad Sci U S A. 2012;109(34):13769-74. doi: 10.1073/pnas.1107435109. PubMed PMID: 22872870; PubMed Central PMCID: PMCPMC3427132.

3. Lundberg DS, Yourstone S, Mieczkowski P, Jones CD, Dangl JL. Practical innovations for high-throughput amplicon sequencing. Nat Methods. 2013;10(10):999-1002. doi: 10.1038/nmeth.2634. PubMed PMID: 23995388.

4. Caporaso JG, Kuczynski J, Stombaugh J, Bittinger K, Bushman FD, Costello EK, et al. QIIME allows analysis of high-throughput community sequencing data. Nat Methods. 2010;7(5):335-6. doi: 10.1038/nmeth.f.303. PubMed PMID: 20383131; PubMed Central PMCID: PMC3156573.

5. DeSantis TZ, Hugenholtz P, Larsen N, Rojas M, Brodie EL, Keller K, et al. Greengenes, a chimera-checked 16S rRNA gene database and workbench compatible with ARB. Appl Environ Microbiol. 2006;72(7):5069-72. doi: 10.1128/AEM.03006-05. PubMed PMID: 16820507; PubMed Central PMCID: PMCPMC1489311.

6. Wickham H. Ggplot2 : elegant graphics for data analysis. New York: Springer; 2009. viii, 212 p. p.
